# Metabolic patterns associated with the seasonal rhythm of seed survival after dehydration in germinated seeds of *Schismus arabicus*

**DOI:** 10.1186/s12870-015-0421-9

**Published:** 2015-02-05

**Authors:** Bing Bai, David Toubiana, Tanya Gendler, Asfaw Degu, Yitzchak Gutterman, Aaron Fait

**Affiliations:** Ben-Gurion University of the Negev, Jacob Blaustein Institutes for Desert Research, French Associates Institute for Agriculture and Biotechnology of Drylands, Midreshet Ben-Gurion, 84990 Israel; Current address: Department of Molecular Plant Physiology, Utrecht University, Utrecht, 3584 CH The Netherlands

**Keywords:** Seed germination, Seed survival, Dehydration, Metabolomics, Annual rhythm

## Abstract

**Background:**

Seed of *Shismus arabicus*, a desert annual, display a seasonal tolerance to dehydration. The occurrence of a metabolic seasonal rhythm and its relation with the fluctuations in seed dehydration tolerance was investigated.

**Results:**

Dry seeds metabolism was the least affected by the season, while the metabolism of germinated and dehydrated seeds exhibit distinct seasonal patterns. Negative associations exist between amino acids, sugars and TCA cycle intermediates and seed survival, while positive relations exist with seed germination. In contrast, associations between the level of secondary metabolites identified in the dehydrated seeds and survival percentage were evenly distributed in positive and negative values, suggesting a functional role of these metabolites in the establishment of seed dehydration tolerance.

**Conclusion:**

Our results indicate the occurrence of metabolic biorhythms in germinating and dehydrating seeds associated with seasonal changes in germination and, more pronouncedly, in seed dehydration tolerance. Increased biosynthesis of protective compounds (polyphenols) in dehydrating seeds during the winter season at the expenses of central metabolites likely contributes to the respective enhanced dehydration tolerance monitored.

**Electronic supplementary material:**

The online version of this article (doi:10.1186/s12870-015-0421-9) contains supplementary material, which is available to authorized users.

## Background

Seed germination is affected by the genetic background and the environmental conditions during seed development and post-dispersal [1,2]. The main factor regulating seed germination is the availability of water, which initiates the seed metabolism through water uptake by rehydrating membranes and oxygenating the inner parts of the seed [3]. Other determinants, such as day length [4], temperature [5] and osmoticum [6] also can modulate seed germination. Germination in arid environments exposes germinated seeds to unpredictable rainfall and prolonged period of drought. Hence, mechanisms for regulation of germination and tolerance to dehydration have evolved determining the degree and timing for germination, a trait, which likely evolved in conjunction with seed survival following dehydration [7]. A significant amount of knowledge has been accumulated on the annual periodicity of the germinability of stored seeds [8-11]. *Arabidopsis* seeds were reported to follow an annual dormancy cycling by an altering sensitivity to the environmental stimulus such as temperature, light and nitrite in different seasons [12,13]. In weed seeds annual dormancy cycles are linked to a continuum of physiological changes possibly related to changes in membrane properties [14] such as the fluidity and membrane protein conformation [15], likely promoting gas exchange in the inner parts of the seed altering its redox state. Reactive oxygen species and nitric oxide were recently suggested to be involved in the regulation of dormancy [16]. Whilst annual periodicity in dormancy of seeds received much attention, more elusive phenomena were shown to be seasonal dependent. For example, *Digitalis purpurea* L. seeds germinated over a period of 13 months under controlled condition were shown to vary in their content of sterol at the same germination stage [17]. The seasonal regulation of sterol was found to correlate with annual cycle in germination likely for the purpose of membrane stabilization and protection during cold winter. More recently a three year study demonstrated the occurrence of annual periodicity in dehydration tolerance of germinated seeds [18]. *Schismus arabicus* Nees (Poaceae), a common fodder in Negev desert, germinated uniformly throughout the year at 80-100%; however the percentage of surviving seed to controlled dehydration experiments varied depending on the season.

Dehydration response in plants involves all levels of cellular activity [19] including metabolic reorganization [20]. For example, the biosynthesis of sugars and polyphenols play a significant role in protein and membrane protection against the effect of dehydration; trehalose, raffinose, galactinol and umbelliferose can promote the formation of protective glass matrix [21-24]; flavonoids can provide a chemical barrier by decreasing permeability to moisture [25] limiting damage during storage [26]; tocopherols - lipophilic antioxidants can limit non-enzymatic lipid oxidation during seed dehydration, storage, and early germination stages [27,28]. Recently metabolite profiling showed the induction of energy metabolism and the biosynthesis of specialized antioxidant as possibly linked with increased germination following dehydration of imbibed Arabidopsis seeds [29].

The aim of the present study was to explore the metabolic basis of seasonal periodicity in seed germination and survival following dehydration in *Shismus arabicus*.

## Methods

*Schismus arabicus* Nees caryopses (seeds) were collected in April 2005 from a natural habitat near Sede Boker in the Negev (34°46′E 30°51′N; 460 m a.s.l). The caryopses were separated and stored in glass vials, placed into brown paper bags and stored at 40°C in darkness controlled with thermostat (Environette, Lab-Line, Illinois, USA) as described earlier [18]. In the current set of experiments only caryopses of the size 350–425 μm were used, which showed to have the highest germination rates and percentage of germination [30].

### Seed germination, dehydration and seed survival measurements

Germination and dehydration experiments were conducted exactly as described in [18]. The experiment started in June 2010 lasting 12 months until May 2011. Briefly, caryopses were germinated in four replicates of 50 caryopses each on wetted (1.5 ml) Fisher No. 1 filter paper vertically positioned under in a vial 55 mm high and 33 mm in diameter. 1.5 ml of distilled water was placed at the bottom of each vial, and the vials were closed and placed at 25°C in darkness. After 24 h of wetting, the average percentage germination was determined. After 24 hours imbibition, the germinated seeds with radicle length of about 0.2-0.3 mm measured by microscope (Olympus SZ61, with scale) were transferred to dry 5 cm diameter Petri dishes and allowed to dry at 25 ± 1°C and 10–15% relative humidity (RH), measured by a thermo-hygrograph throughout the sets of experiments. Following 180 min dehydrated germinated seeds were stored in the same conditions for 21 days. After the period of dry storage, the filter papers with the dehydrated seeds were placed on petri dishes and re-wetted with 1.5 ml water. The closed petri dishes were stored first in darkness at 15°C for 48 h, and then at 15°C under low light of 100 μmol m^−2^ s^−1^. Seeds were scored as “survived” when both root and coleoptile elongation continued after 21-d rehydration (Additional file 1c).

### Extraction for the identification and quantification of metabolites

50 dry caryopses, germinated seeds and dehydrated seeds per replicate were extracted for parallel metabolite profiling as described in [31]. Seeds were homogenized using previously cooled mortar and pestle with liquid nitrogen and extracted in a pre-chilled methanol:chloroform:water extraction solution (1:2.5:1 v/v) for 30 min at 4°C shaking. Standards, i.e. 0.2 mg/ml ribitol, 1 mg/ml ampicillin in water, 1 mg/ml corticosterone in methanol and 5 mg/ml heptadecanoic acid in chloroform, were subsequently added. After centrifugation at 2,200 g, the remaining pellet was extracted in a second step with 500 μl methanol/chloroform. The extracts were combined and 500 μl of water was added to the supernatant to separate the chloroform phase from the water/methanol phase. The latter was used for metabolite analysis via GC-MS DSQII (Thermo-Fisher ltd.) and UPLC-Xevo-QTOF-MSMS (Waters ltd) exactly as described in [29]. A volume of 200 μl of water/methanol extract was reduced to dryness in vacuum. Residues were derivatized and analyzed via an established GC-MS based method adapted to seeds [32]. GC-MS data were processed by Xcalibur® and normalized by the internal standard ribitol. The UPLC raw data were recorded with the aid of MassLynx version 4.1 software (Waters ltd). Metabolites were identified by using MassLynx software and searched against metabolite database Chemspider (http://www.chemspider.com/). The quantification of the compounds is based on the relative peak response area of each mass signal after pareto scaling in the chromatograms and normalized to the tissue DW.

### Statistical analysis

The significance between the germination percentage of the caryopses and percentage of seeds that survived was tested by one-way ANOVA following arcsin transformation. Principal component analysis (PCA), t-test and ANOVA were implemented using the software TMEV [33]. The term significant is used in the text for p-values lower than 0.05 (p < 0.05).

### Network analysis

The coordinated behavior of metabolites can be delineated using graph theory, where the nodes represent metabolites and the relationship between them is demonstrated as edges. The generation of the graphs was based on the correlation analysis of all metabolites and the two physiological traits (germination and survival percentage). Prior to correlation analysis, each metabolite was normalized by its respective mean calculated across the time-point measurements. Physiological traits were arcsin transformed. In addition each component (metabolites and physiological traits) of the dataset was pareto-scaled. Normal distribution was tested across all time-points by employing a Shapiro-Wilk test. In most cases (dry seed network = 74.0%, germination network = 92.2%, dehydration network = 79.2%) the assumption of normal distribution was violated. Thus, the non-parametric Spearman’s rank correlation was chosen over the parametric Pearson correlation to compute correlation coefficients.

To reconstruct a network capturing coordinated changes in metabolic and physiology profiles, first the corresponding p-value threshold Spearman rank correlation coefficient ensuring a *q*-value of 0.05 was determined. Second, the adequate correlation coefficient threshold was chosen by assessing four different network properties, i.e. average node degree, clustering coefficient, network density, and diameter. For a full description on these network properties the reader is referred to [34]. The correlation coefficient, at which the network displayed a robust behavior across a range of p-values in all four properties, was chosen as the threshold for network construction. Subsequently, the network was analyzed for communities by employing the walk trap community algorithm [35]. The significance of the communities with more than nine nodes was tested by performing a Wilcoxon signed rank test. The test was performed by assessing the degree of node-connectivity [34] of the isolated community as compared to the nodes of the community still embedded in the network of which all community specific edges have been subtracted.

All computations for network visualizations were generated in the R environment. The software Cytoscape [36] version 2.8.3 was used for network visualization itself. Network properties and communities were computed by using the igraph R package (Additional file 2).

## Results

### Seed germination and recovery after rehydration

**Figure 1:**
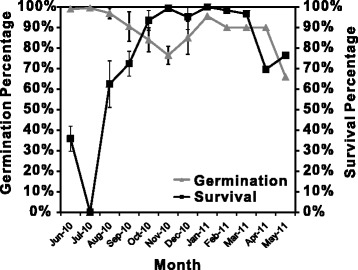
**Monthly changes in seed germination percentage and seed survival.** Seed germination percentage was measured following 24 hours of imbibition; survival percentage was measured following a three-week dehydration of the germinated seeds.

Germination and seed survival percentage during the 2010–2011 experiment closely reflected the values measured in a previous three-year study conducted on seeds from a different harvest [18]. In detail, germination percentage of *S. arabicus* caryopses from June to September 2010 was above 90% with a decreasing trend in November to 76.5 ± 2.2% (Figure 1). From December 2010 to April 2011, the percentage of germination was kept at about 90%, followed by a significant drop in May to 66.0 ± 4.3%. This relatively stable germination percentage contrasted drastically the changes scored for seed survival following dehydration. Seed survival dropped to 0% in July 2010 and peaked during the month of January 2011 with 100% recovery in all four independent preparations with 50 seeds each (Figure 1). The seasonal survival displayed similar patterns to the meteorological data as indicated in (Figure 1 and Additional file 3), obtained from the meteorological station at the Jacob Blaustein Institutes for Desert Research of the Ben Gurion University of the Negev (http://www.bgu.ac.il/BIDR/research/phys/meteorology/). Soil and air temperature, global radiation and rainfall are all following a seasonal cycle, characterized by the high temperature, more intensive radiation and complete lack of rainfall during the summer and by colder temperatures, reduced radiation and low rainfall during the winter (Additional file 3).

### Seed germination and dehydration are characterized by season-specific metabolite profiles

**Figure 2:**
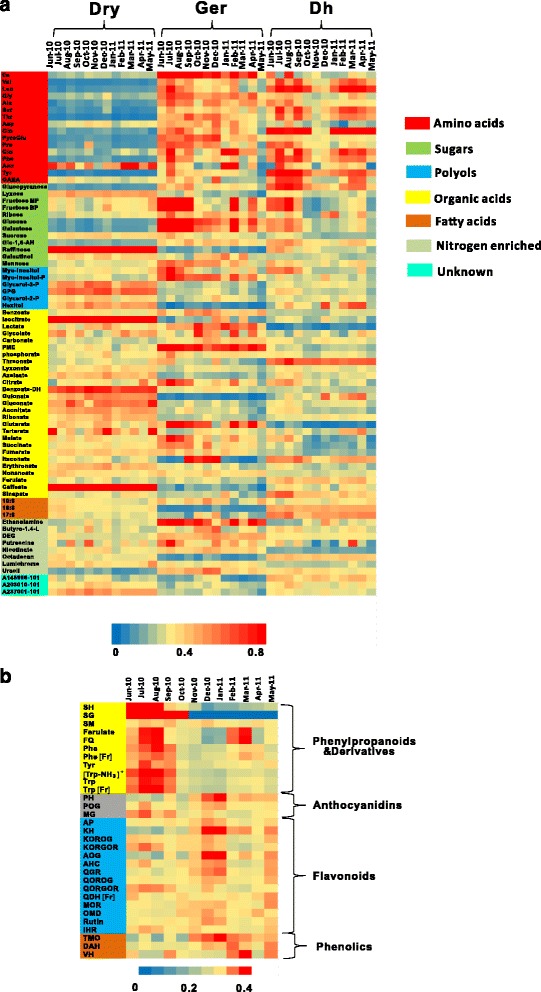
**Heatmap visualization of primary metabolic profile of dry seeds (Dry), germinated (Ger) and dehydrated seeds (Dh) (a) and secondary metabolic profile of dehydrated seeds (b) through the year.** The value of each metabolite entry was scaled between 0–1 as indicated by the color scale in the heatmap.

**Figure 3:**
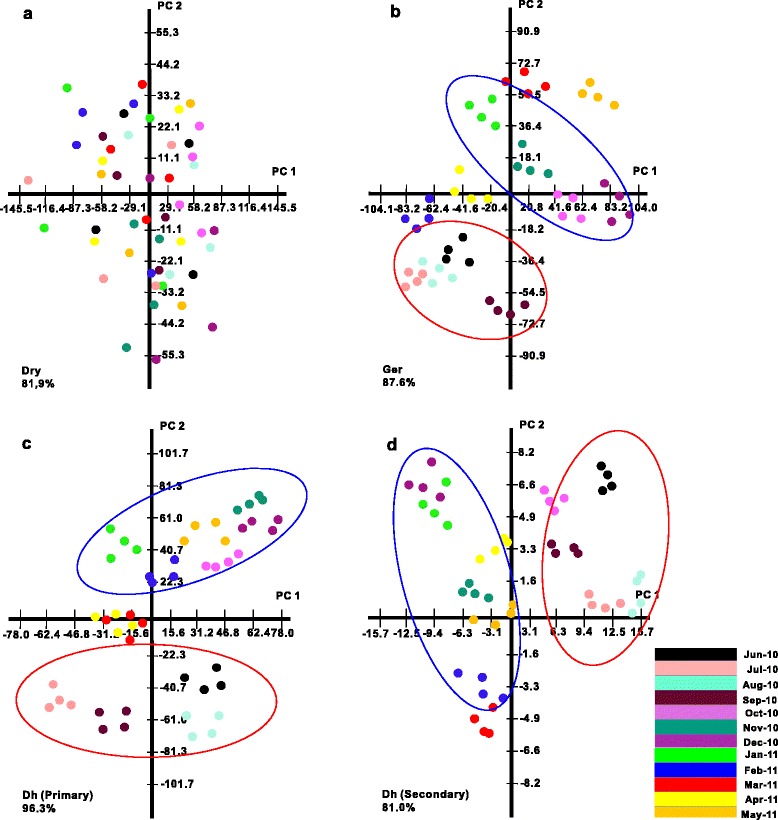
**Principal Component Analysis (PCA) of seasonal effect on primary metabolite content of dry seeds (a), germinated seeds (b) and dehydrated seeds (c) and on secondary metabolites content of dehydrated seeds (d) through the year.** The percentage of total variation explained by the first two principal components are shown. The separation of summer and winter is shown in red and blue ellipses.

Metabolite profiles of central metabolism of dry, germinated and dehydrated seeds were generated (Figure 2). Principal Component Analysis (PCA) was conducted on the year-long metabolite dataset to investigate the relative impact of single metabolites and seasonal changes on metabolic shift (Figure 3 and Additional file 4). In the dry seeds, monthly metabolic changes were minor, leading to no visible separation between monthly profiles (Figure 3a and Additional file 5). In contrast, germination on a monthly resolution was associated by changes in the metabolic profiles of the germinated seeds across the year. For example, notable is the separation on the 1st component of the “summer” samples (June-September) from the winter samples (October-January) (Figure 3b). Monthly dehydrated seeds also displayed a seasonal metabolite profiles in a similar but more accentuated manner than germinated seeds. Summer (June, July, August and September 2010) and winter months (November and December 2010, January and February 2011) could be distinguished on PC2 (Figure 3c). Same seasonal separation following dehydration was shown for secondary metabolites by PCA (Figure 3d).

### Metabolic changes during *Schismus* seed germination and dehydration

First we investigated the common changes in metabolite profiles during germination and dehydration, independent of the season. Central and specialized metabolites followed generally conserved patterns during germination and dehydration (Figure 2 and Additional file 6a and b). As expected, seed germination was characterized by enhanced carbon - nitrogen metabolism featured by the accumulation of glucose, glucopyranose and fructose at the expenses of sucrose, the accumulation of glutamine, pyroglutamate and proline together with most amino acids, with the exception of asparagine. The decrease of aconitate and isocitrate associated to the TCA cycle was coupled to the accumulation of 2-oxoglutarate, succinate and malate. Free fatty acids and associated glycerol derivatives dropped in content in germinated seeds compared to dry ones, suggesting a role in supporting early germinative processes. Cell wall associated metabolites glucoronate, gulonate and lyxose decreased sharply during seed germination, in contrast to a 23 fold change (FC) accumulation of glucose, a 17 FC accumulation of galactose and a two fold increase of mannose, suggesting an expected repartitioning of C metabolism. Raffinose drastically decreased in content upon imbibition validating its role as transient C storage molecule as suggested earlier [37,38]. The shikimate derived and precursors of the phenylpropanoid pathway, phenylalanine (5.8 FC) and tyrosine (9.8 FC) accumulated in the germinated seeds, but not so the derived caffeate and ferulate (Additional files 5 and 6a).

Dehydration of germinated seeds resulted in attenuated effect on the abundance of most intermediates of the central metabolism (Additional file 5 and 6b). Notable was the accumulation of the non-proteinogenic amino acid GABA (1.8 FC), hydrophobic branched chain amino acids glutamine (12 FC), valine (1.6 FC), leucine (2.8 FC), and serine (2.2 FC) and shikimate derived phenylalanine (1.6 FC) and tyrosine (3.0 FC). Also ascorbate precursor galactose and derived threonate, raffinose and pentose phosphate pathway intermediates gluconate, gulonate and lyxonate accumulated during dehydration. Dehydration induced the activation of the phenylpropanoid pathway reflected by the accumulation of precursor amino acids and representative phenylpropanoids sinapate, caffeate and ferulate (Figure 2 and Additional files 5 and 6b).

### Seasonal impact on germinated and dehydrated seeds

Germination percentage in the summer and winter were relatively stable (Figure 1), while the metabolism of germinated seeds displayed a seasonal pattern (Additional files 6c and 7). In the summer, a significant (p < 0.05) higher abundance was observed in glycolysis intermediates glucose, fructose and intermediates associated with energy production succinate, fumarate and malate in TCA cycles as compared with the seeds germinated in the winter months. The latter were instead characterized by relatively higher itaconate, tartarate, glycolate, glycerol derivatives and identified fatty acids, and also by significantly higher level of intermediates of the pentose phosphate pathway, in contrast to 1/4 the content of myo-inositol. Seeds germinated in the winter showed also accumulation of phenolic compounds ferulate, sinapate and “protective” sugars such as galactinol and to a lesser extent raffinose and Additional file 5.

Seasonal changes significantly affected the seed survival percentage following dehydration, which was measured at an average of 42.7% in the summer compared with 98.2% in the winter (Additional file 8).

Metabolite profiles on dehydrated seeds during the season (Additional file 6d) generally followed a similar pattern to the one observed in germinated seeds. Hence the identified shifts in metabolism in germinated seeds from summer to winter might be functional to seed survival upon dehydration. In the summer, the carbon pool, particularly of the sugars and TCA cycle intermediates was greater than in the winter, suggesting a lower carbon partitioning rate (Additional file 6d). Dehydrated seeds showed a seasonal trend in the free pool of amino acids being greater in the summer. Similarly to central metabolism, secondary metabolites showed seasonal specific profiles (Figure 2b, Additional files 6d and 7). Ten phenylpropanoids were accumulated mainly in the summer together with the two aromatic amino acids phenylalanine and tyrosine (Figure 2b). However, down-stream phenylpropanoid derived phenolic compound flavonoids (thirteen out of fourteen) and anthocyanins (all three) detected were higher in winter season, suggesting a seasonal dependent regulation of the biochemical steps linking the higher and lower portion of the phenylpropanoid pathway.

Interestingly, putatively identified 1-O-sinapoyl-β-D-glucose was detectable following dehydration only during the summer from June to October, months characterized by the lowest seed dehydration tolerance.

### Network analysis sheds insights into the relation between germination, survival percentage and metabolism

In an attempt to understand the coordinated metabolic shifts characterizing dry seeds, germinated seeds and dehydrated seeds across the year and in order to identify key metabolites associated with seed tolerance to dehydration, we employed correlation-based network analysis (CNA). Within the CN we included the relationships between the physiological traits (germination and survival percentage) and metabolites.

The metabolic network of the dried seed (Additional file 9a) is composed of four main communities, of which communities 1 and 3 are significant in respect to the community affiliation (Comm. 1 – p = 1.58e-09, Comm. 2 – p = 0.821, Comm. 3 – p = 0.009, Comm. 4 – p = 0.085). Comm. 1 incorporates all represented compound classes and is characterized by a high degree of positive correlations and homogenous nodal degree (number of links per node). The most profound feature of this network is the few relations with the physiological traits tested. The germination percentage correlates solely, and negatively, to gulonate while, the seed survival correlates negatively to the germination percentage, a feature suggesting that overall in periods of low germinability the seeds that do germinate have a high probability of tolerating the dehydration process. On the other hand in periods of high germinability the percentage of survival is not a prominent feature.

The metabolic network associated with germination (Additional file 9b) also displays four main communities. However, the structure of the communities reveals notable differences. Community 3 and 4 of the dry-seed metabolite network are not present in the germinated network emphasizing the shifts that undergo amino acid and energy related metabolism from the dry seed to germination. The current view reveals two main communities, which integrate a similar number of nodes (Comm. 1 = 21 nodes and Comm. 2 = 23 nodes), including most amino acids and sugars present in the network. Furthermore, Comm. 1 integrates the two physiological traits indicating that the profiles tested for each correlates significantly to the metabolic profiles measured during germination. Taken together, these data and the chronological order of events suggest that the processes during germination are primary factors affecting seed tolerance to dehydration.

The dehydration metabolite network (Additional file 9c) revealed the occurrence of two main communities. The most salient community (p-value of 1.82e-14) shown is the densely intra-connected community 1, incorporating 40 of the 71 nodes and exemplifying the highly coordinated shift across the year taking place in dehydrated seeds. This community entails the entire array of compound classes represented, as well as the two physiological traits, seed germination and seed survival, suggesting that the germination percentage and seed dehydration tolerance across the year are in part the expression of a defined and coordinated shifts in the metabolic phenotype.

**Figure 4:**
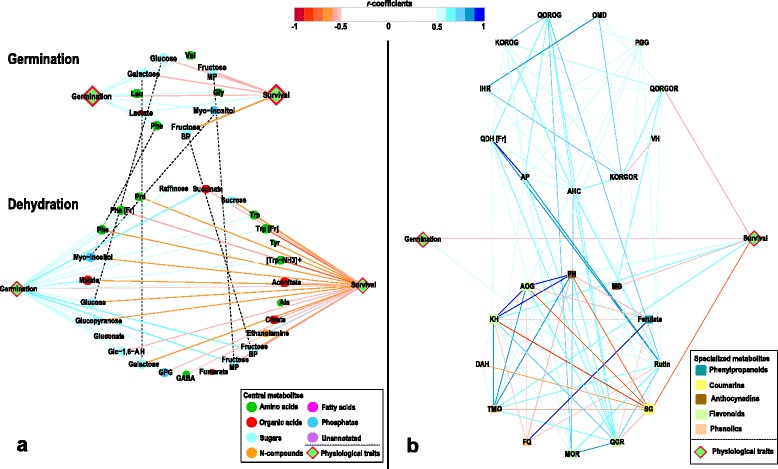
**Integrated network for the association of central (a) and specialized metabolites (b) with seed germination and seed survival percentage.** Nodes in the network are color-coded according to their compound classes and shaped according to their specificity (elliptical = central metabolism, rounded rectangles = specialized metabolism, diamond shaped = physiological traits). Relative sizes of nodes correspond to their degree of connectivity. The Spearman rank correlation was employed to compute all pairwise correlations between metabolites across the timeline. Solely significant correlations were chosen to be depicted. A significance level of q < 0.05 and an r-value of >0.5 were considered to be significant.

To further investigate the relationship of survival and germination with the metabolite profiles changes during the year, isolated subgraphs integrating solely the adjacent nodes were generated (Figure 4). The commonalities between germination metabolism and dehydration metabolism within the subgraphs were highlighted by dashed lines. The germination percentage correlates positively to all compounds connected, whilst- generally - survival percentage correlates negatively to the same compounds, i.e. the sugars fructose, glucose, and the cell wall associated sugar galactose as well as the sugar alcohol myo-inositol in both networks. Specifically, when dehydrated seeds are low in intermediates of the glycolysis and TCA cycle, the corresponding survival percentage is relatively high; a similar trend is observed for the amino acids for the shikimate pathway, and stress related Pro, GABA and Ala. Outstanding is the positive relation between the content of ethanolamine in the dehydrated seeds and their survival. When the yearly metabolite profiles of the dehydrated seeds was subjected to network analysis, metabolite 1-O-sinapoyl-β-D-glucose highly correlated with another putatively identified compound sinapic acid hexose (SH, r = 0.85, p = 0.0004) and with its precursor sinapate(r = 0.77, p = 0.0033), however low correlation was found with the down-stream metabolite sinapoyl malate (SM, r = 0.49, p = 0.1034). It also displayed the strongest negative connection with seed survival and also negatively correlated with other metabolite cluster such as flavonoid and anthocyanidin, especially four nodes pelargonidin hexose (PH), Apigenin-7-O-glucoside (AOG), kaempferol hexose (KH) and pentanoic acid (TMO), which are strongly positively interconnected. In contrast to the central metabolites, several of the secondary metabolites in the dehydrated seed**s** positively related with their survival percentage, TMO, KH, AOG and PH.

## Discussion

Seed germination displaying annual rhythm was the focus of several studies and shown to be at least in part under endogenous regulators [39]. However a significant gap in knowledge exists in respect to the metabolic processes associated with germination rhythms. Here we presented the first evidence of seasonal metabolic fluctuations in germinated seeds and dehydrated seeds and its relation to germination and seed survival following dehydration. In spite of relatively stable germination across the year, the number of seeds of *S. arabicus* that could survive three weeks dehydration was largely affected by the annual periodicity of dehydration treatment. Namely seeds survived dehydration in the winter season from October to March with almost 100% survival, in contrast to the low survival in the summer from April to September and consistent with previous reports. Neither germination, nor seed survival were found to be associated with the metabolism of the dry seed, which displayed a uniform profile throughout the year. These results suggest a distinct regulation mechanism of seasonal changes in dehydration tolerance of *S. arabicus* compared with dormancy cycling in *Arabidopsis* dry seeds, which is attributed to the integration of the molecular physiological state with changes in sensitivity to the environment [8,12,40]. The endogenous rhythm of *S. arabicus* is presumably set already in advance during seed development. An endogenous molecular clock is likely stored in the form of mRNAs and epigenetic phenomena [41,42]. During seed imbibition, water intake activates the cellular metabolism by enhancing enzymes activities associated storage reserve mobilization, hormone mobilization and seed respiration [43,44]. These processes are reflected in seeds by decreased sucrose content and concurrent increase of glycolysis and TCA intermediates and fatty acids, and by the conversion of Asn to Asp, as shown in this study and as previously reported in *Arabidopsis* [38]. During seed germination, also the flavonoid biosynthetic pathway is induced as shown by the accumulation of shikimate derived Phe and Tyr precursors of the phenylpropanoid pathway, and the reduction of caffeate and ferulate could suggest for enhanced integration within downstream processes. Upon dehydration, stress related metabolic processes are induced [45,46] including the accumulation of GABA, branched chain amino acids Val and Leu, raffinose and galactose and phenylpropanoids [47].

### Seasonal rhythm affects stress related metabolism linking to seed germination and seed survival

Seed germination and seed survival are negatively related (Figure 1 and Figure 4a) suggesting that during periods of low germination, those seeds that do germinate will eventually tolerate dehydration. Is germination the period of priming for seasonal changes in seed tolerance? Metabolite profiling and network analysis suggest that, for central metabolism, namely this might be the case. The experiments were conducted in controlled conditions, where water, temperature and light during germination were at constant levels throughout the season. Nevertheless, germinated seeds in the summer show a very different metabolite profile compared to the winter, characterized by increased amino acids content, accumulation of primary sugars, TCA cycle and shikimate intermediates. A characteristic of the winter, imbibed seeds (and better germinating) was a general lower content of metabolites throughout the profile, except for the accumulation of itaconate, and glycolate. These results might suggest a higher metabolic turnover.

When gradually dehydrated, seeds accumulated amino acids, sugars and fatty acids particularly in the summer likely dedicated to the formation of a glassy matrix to counter the loss of water [48,49]. Network analysis could differentiate those metabolites jointly associated with trends in germination and dehydration tolerance from those specific to dehydration and likely more relevant to seed survival (Figure 4), e.g. sucrose, TCA cycle intermediates and ethanolamine accumulation. The annual rhythm of seed survival was also associated with the accumulation of phenylpropanoid precursors of the shikimate pathway in the summer and of downstream compounds, including kaempferol, quercetin and their derivatives in the winter. These flavonoids have been long recorded to be involved in wounding response, pathogen plant interaction and provide protection from irradiation and UV [50-54]. These results suggest for an enhanced capacity of the winter-seeds to repartition the C pool and accumulate protective compounds, especially in the polyphenol group, thus reducing detrimental cellular damages. A compound, tentatively identified as 1-O-sinapoyl-β-D-glucose, was present at detectable level from June to October only, i.e. the summer period. Its significant correlation with the precursor sinapate and low correlation with the down-stream product sinapoyl malate indicates that the turnover of sinapate could be a potential marker for dehydration tolerance.

No significant differences were detected in membrane permeability between seasons (Additional file 10). Hence we can safely conclude that the differences encountered are not due to impaired oxygen diffusion in the inner parts of the seed during the summer or differences in the membrane stability. Nevertheless we cannot exclude the occurrence of other processes within the dry seed that might affect the seasonal differences in dehydration tolerance.

## Conclusion

By employing seeds of a desert annual, *Schismus arabicus*, metabolic profiling of dry seeds, germinated and dehydrated seeds revealed metabolic features closely associated with the documented annual rhythm of seed survival. Overall metabolite profiling and network analysis show that metabolic processes during germination seem to characterize the degree of seed dehydration tolerance. In the summer, the accumulation of central metabolites during germination, likely from a lower turnover, and a lower content of “protective” compounds could contribute to the lower tolerance of the seed to dehydration. The existence of inhibitory compounds accumulating during the summer, e.g. 1-O-sinapoyl-β-D-glucose, should be further investigated. In addition, future studies shall investigate the regulatory processes involved in the metabolic and physiological patterns here characterized including the occurrence of associated epigenetic phenomena during seed development.

### Availability of supporting data

The data sets supporting the results of this article are included within the article and its additional files.

## Additional files

Additional file 1:
***Schismus arabicus*** **Nees caryopses (seeds) were collected in April 2005 from a natural habitat near Sede Boker in the Negev and the seeds around 425 mm were selected for the experiment.** Dry seeds were aligned on the filter paper (a) followed by 24 hours imbibition (b). Then the germinated seeds were subjected to controlled drying for 21 days and rehydrated by reapplying water. Following rehydration, seeds were scored as viable based on their ability to reestablish root, coleoptile and a continuation of coleoptile elongation (c). Additional file 2:
**Network properties.** Listed are network properties, corresponding to the networks in Figure 4 and Additional file 9, used to determine the significant edges shown in the networks. Additional files 3:
**Meteorological data in the Sede Boqer area between 2010 and 2011.** The data were obtained and summarized from the meteorological station at the Institutes for Desert Research Midreshet Ben Gurion, Sede Boqer. Additional file 4:
**Metabolite loading of dry seeds (DRY), germinated seeds (GER) and dehydrated seeds (DH) in PCA plot (Figure** 3 **).** The loading value of first three principal components are shown. Additional file 5:
**t-test (p=0.05) of detected metabolites in different developmental stages and with seasonal effect.** Values following each metabolite are p value, −log10(−p) and false discovery rate (FDR) of each compared group which are germinated seed compared with dry seeds (GER&DRY), dehydrated seeds compared with germinated seeds (DH&GER), and their respective fold changes under seasonal effect represented by the ratio between winter and summer during germination (WIN&SUM GER) and dehydration (WIN&SUM DH). Additional file 6:
**Schematic view of metabolites enrichment during seed germination (a) and dehydration (b) and the temporal distribution of metabolites of imbibed seeds (c) and dehydrated seeds (d) in different seasons.** Jun, Jul, Aug and Sep were selected as the representative of summer months and Nov, Dec, Jan and Feb were selected as the representatives of winter month. Standard paired t-test was used to compare the metabolite content in germinated seeds with dry seed and dehydrated seeds with germinated seeds in each month and standard unpaired t-test was performed to compare the metabolites significantly enriched in each season. Red and blue circles represent increase or decrease, respectively, in metabolite abundance during seed germination (a) or dehydration (b), in summer (c) and winter (d), p=0.05. Additional file 7:
**Fold changes of detected metabolites in different developmental stages and with seasonal effect.** Value for each metabolite mean fold change (FC) of three replicates of each compared group which are germinated seed compared with dry seeds (GER&DRY), dehydrated seeds compared with germinated seeds (DH&GER), and their respective fold changes under seasonal effect represented by the ratio between winter and summer during germination (WIN&SUM GER) and dehydration (WIN&SUM DH). Additional file 8:
**The average germination and seed survival percentage following dehydration in summer and winter.** *p=0.05, **p=0.01. Additional file 9:
**Network visualization of metabolites as analyzed on dry** ***Shismus arabicus*** **seeds (a), germinated seeds (b), dehydrated seeds (c).** Metabolites are clustered according to the walktrap community algorithm. Positive correlations are denoted as blue edges, negative correlations are denoted as red edges. The sizes of the nodes represent the relative degree of connectivity, The widths of edges in the network correspond to the relative magnitude of correlation estimated. Additional file 10:
**The dry seed leakage conductivity during the summer and winter months.**

## Abbreviations

DEG: Diethylenglycol
Benzoate DH: Benzoic acid, 3, 4-dihydroxy PME, Phosphoratemonomethyl ester
PyroGlu: Pyroglutamate
GPG: Glycerophosphoglycerol
SH: Sinapic acid hexose
SG: 1-O-sinapoyl-β-D-glucose
SM: Sinapoyl malate
Phe: Phenylalanine
Phe [Fr]: Phenylalanine Fragment
Tyr: Tyrosine
Trp: Tryptophan
Trp [Fr]: Tryptophan Fragment
PH: Pelargonidin hexose
POG: Peonidin 3-O-glucoside
MG: Malvidin-3-glucoside
AP: Artonin P
KH: Kaempferol hexose
KOROG: Kaempferolerol-3-O-rutinoside-7-O-glucoside
KORGOR: Kaempferol-3-O-a-L-rhamnopyranosyl(1,2)-b-D-glucopyranoside-7-O-a-L-rhamnopyranoside
AOG: Apigenin-7-O-glucoside
AHC: Apigenin-C-hexoside
QGR: Quercetinrcetin-glucose-rhamnose
QOROG: Quercetin 3-O-rutinoside-7-O-glucoside
QORGOR: Quercetin-3-O-a-L-rhamnopyranosyl(1,2)-b-D-glucopyranoside-7-O-a-L rhamnopyranoside
QDH [Fr]: Quercetin-deoxyhexoside-hexoside fragment
MOR: 3-Methylquercetin 3-O-rutinoside
OMD: O-methylquercetin-deoxyhexoside
IHR: Isorhamnetin-Hex-Rha
TMO: (S)-2-(3-(4-hydroxyphenethoxy)-4-nitrobenzamido)-5(methylthio) pentanoic acid
FQ: Feruloylquinic acid
DAH: Dihydroxybenzoic acid hexoside
VH: Dihydroxy-methyl-benzoic acid hexoside (vanillic acid hexoside)
