# Supplementary figures and images for: Metabolic patterns associated with the seasonal rhythm of seed survival after dehydration in germinated seeds of Schismus arabicus
Source: BMC Plant Biol. 2015 Feb 5;15:37. doi: 10.1186/s12870-015-0421-9 (PMC4330942; doi:10.1186/s12870-015-0421-9)

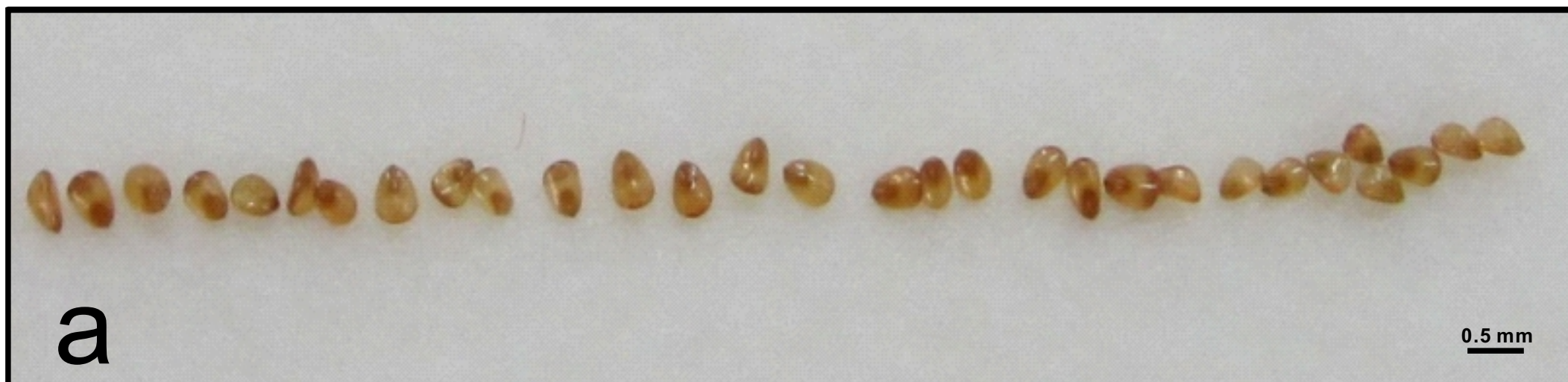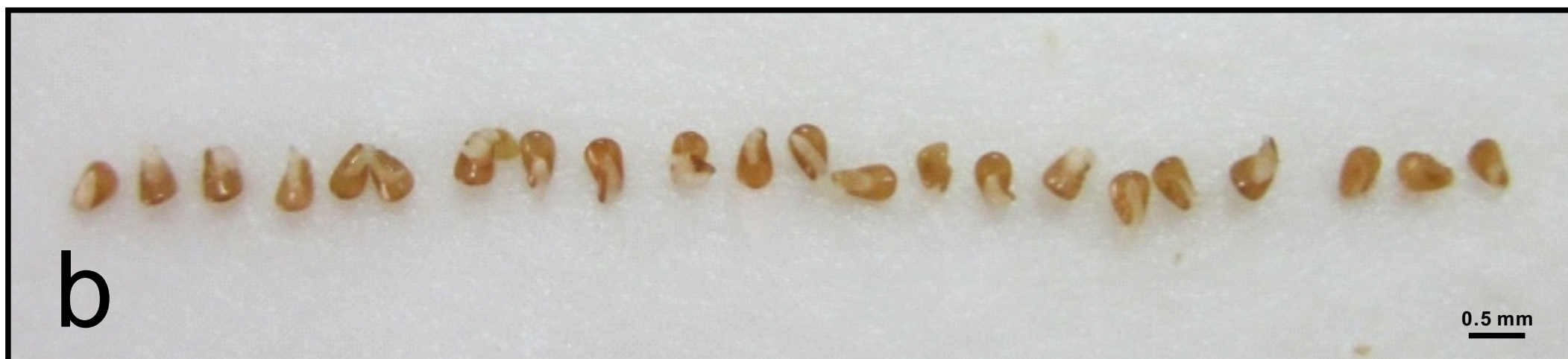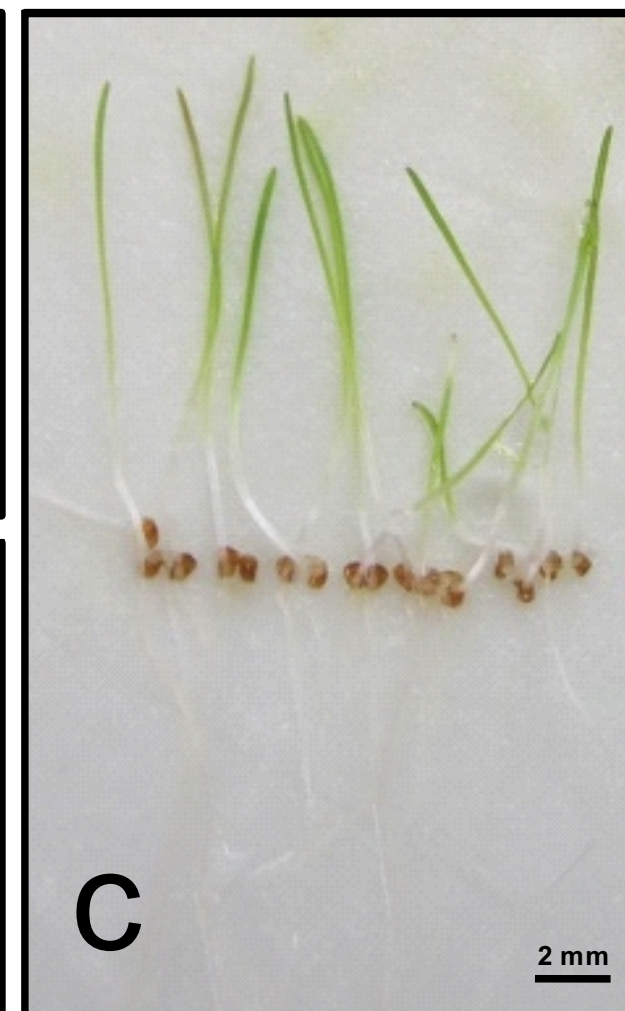

Supplement: Additional file 1: — Schismus arabicus Nees caryopses (seeds) were collected in April 2005 from a natural habitat near Sede Boker in the Negev and the seeds around 425 mm were selected for the experiment. Dry seeds were aligned on the filter paper (a) followed by 24 hours imbibition (b). Then the germinated seeds were subjected to controlled drying for 21 days and rehydrated by reapplying water. Following rehydration, seeds were scored as viable based on their ability to reestablish root, coleoptile and a continuation of coleoptile elongation (c). [file 12870_2015_421_MOESM1_ESM.pdf]

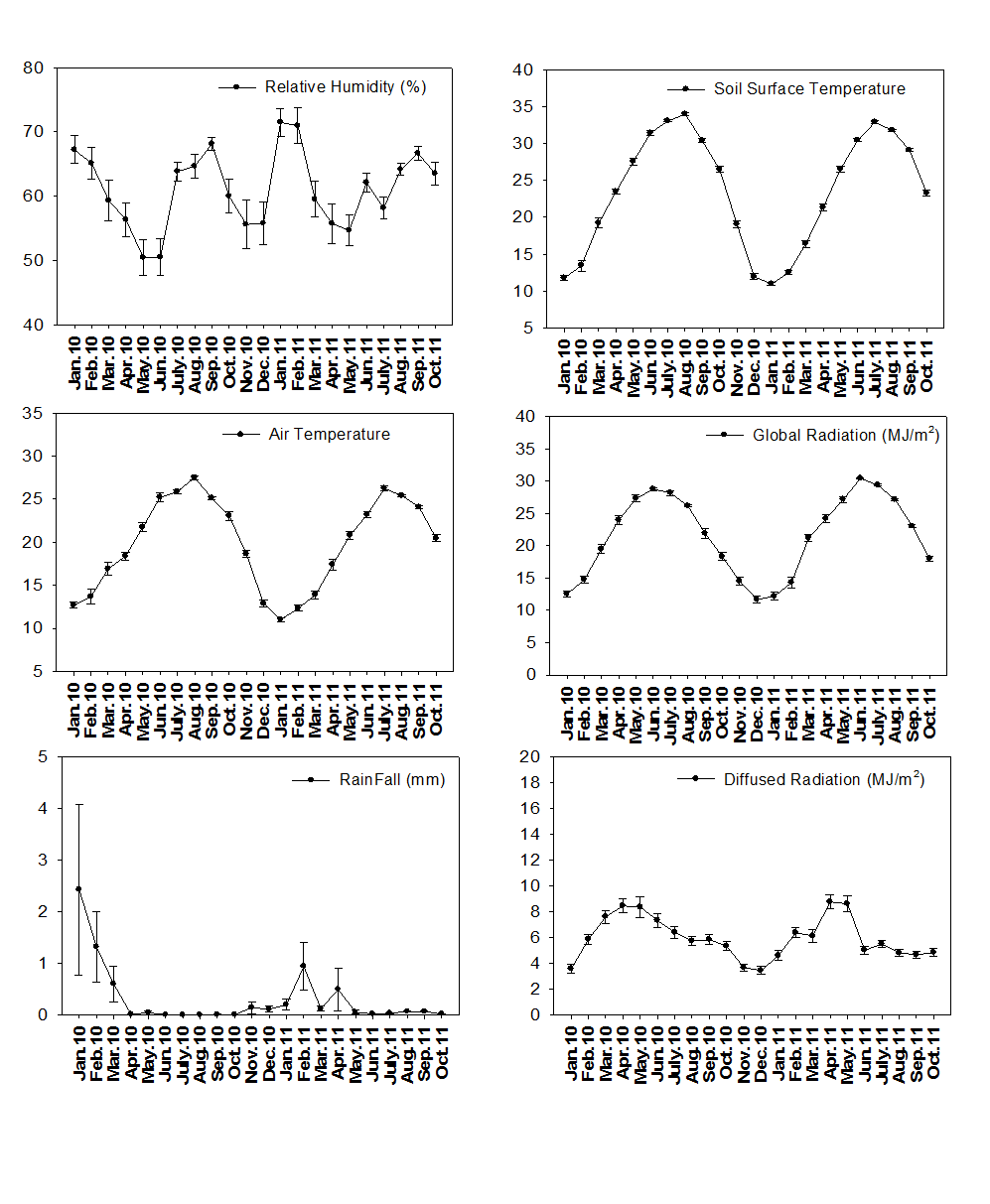

Supplement: Additional files 3: — Meteorological data in the Sede Boqer area between 2010 and 2011. The data were obtained and summarized from the meteorological station at the Institutes for Desert Research Midreshet Ben Gurion, Sede Boqer. [file 12870_2015_421_MOESM3_ESM.tiff]

a

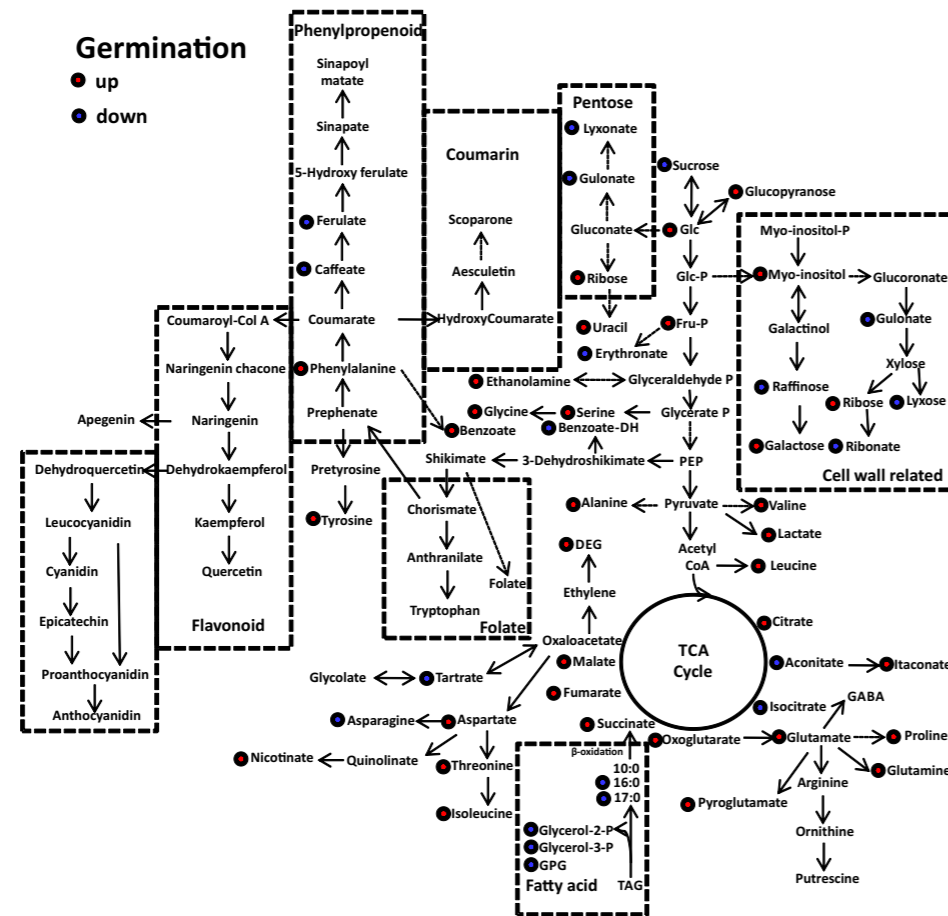

c

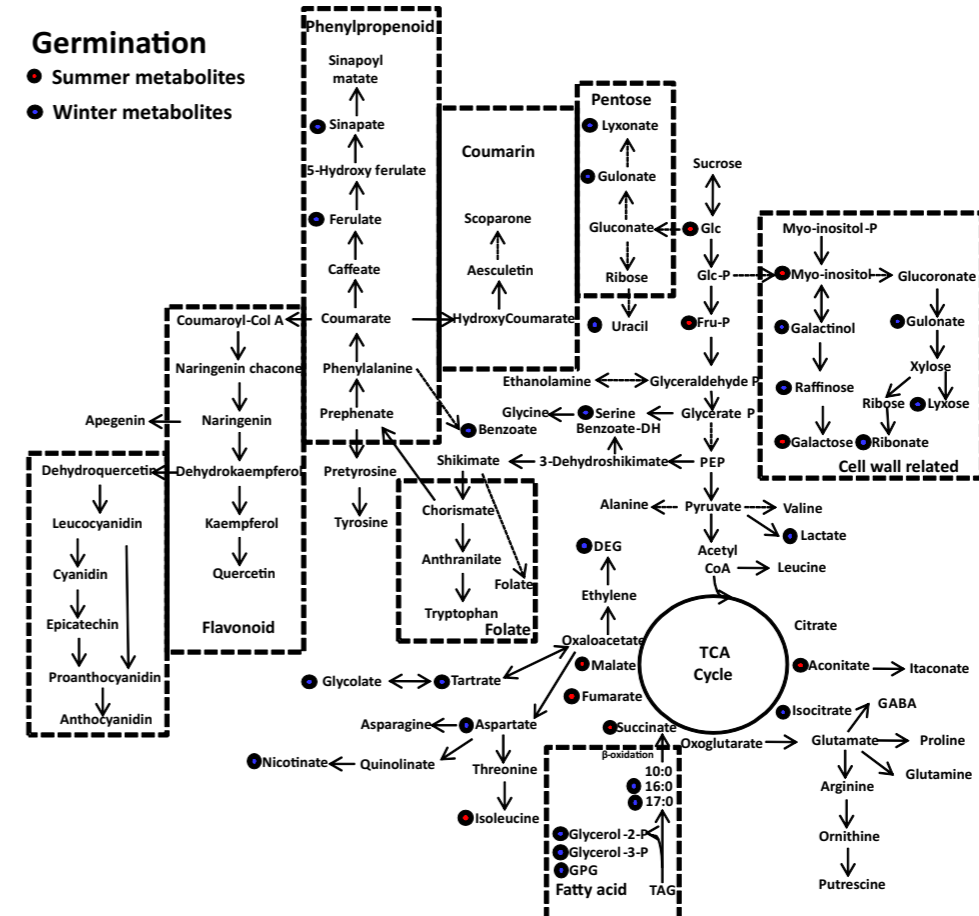

b

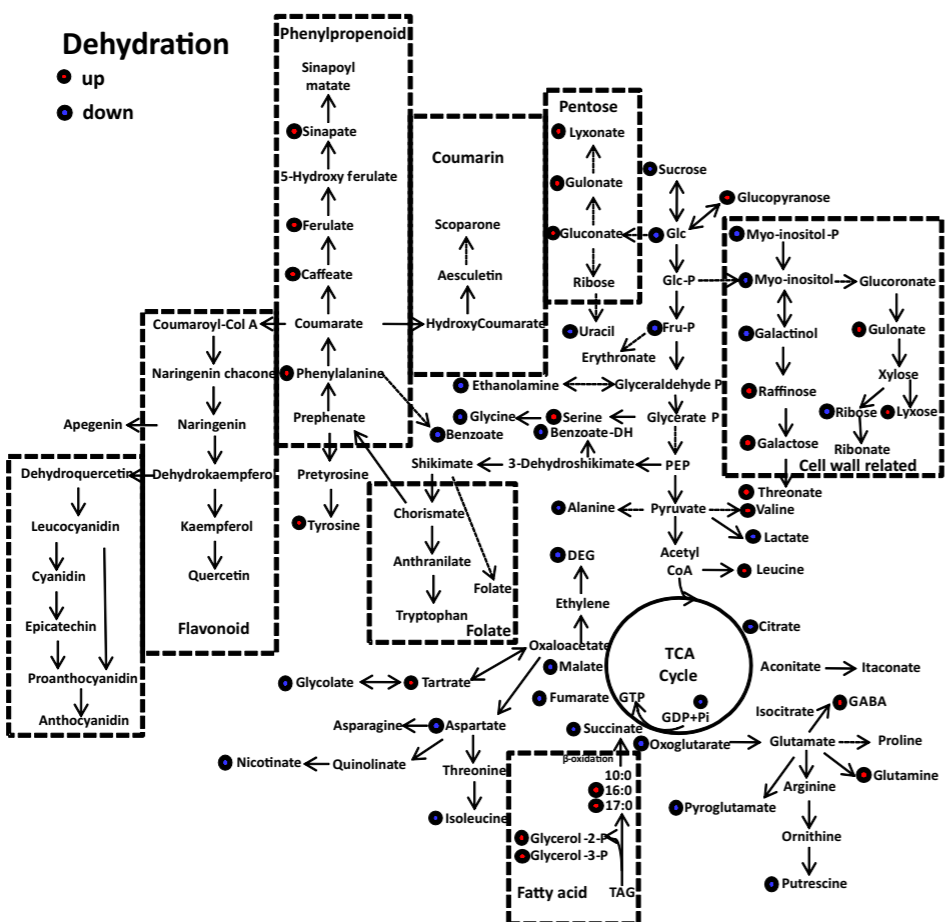

d

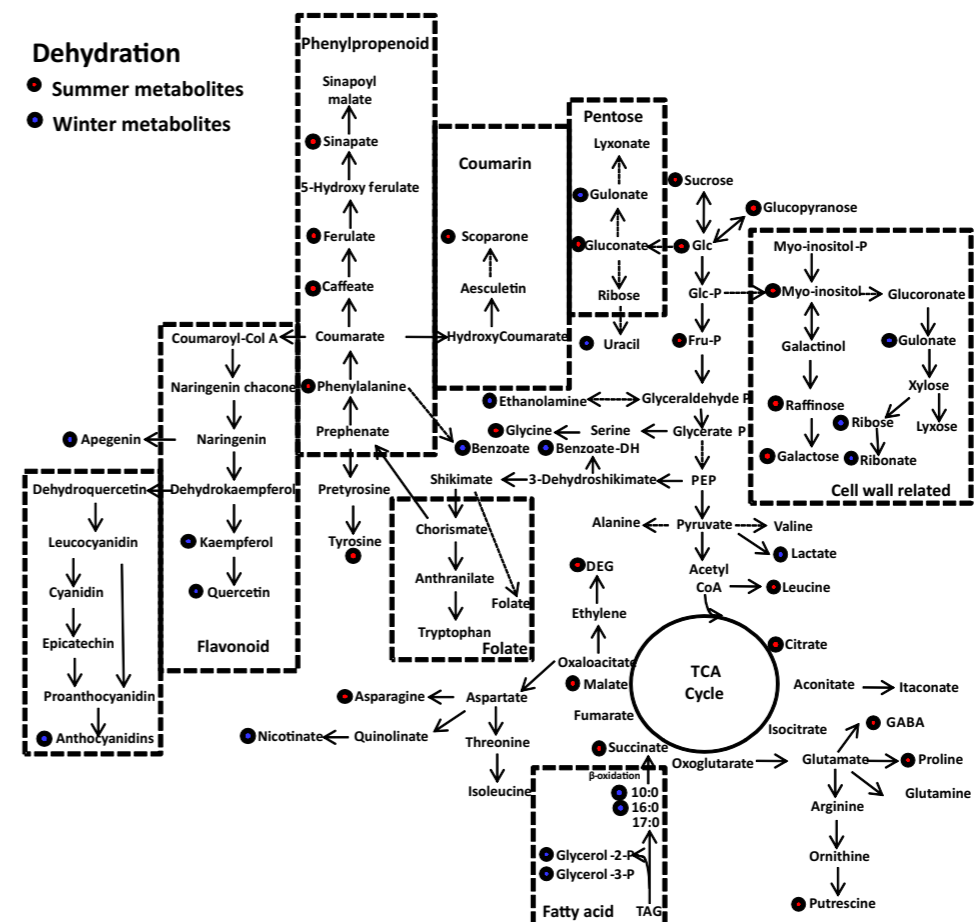

Supplement: Additional file 6: — Schematic view of metabolites enrichment during seed germination (a) and dehydration (b) and the temporal distribution of metabolites of imbibed seeds (c) and dehydrated seeds (d) in different seasons. Jun, Jul, Aug and Sep were selected as the representative of summer months and Nov, Dec, Jan and Feb were selected as the representatives of winter month. Standard paired t-test was used to compare the metabolite content in germinated seeds with dry seed and dehydrated seeds with germinated seeds in each month and standard unpaired t-test was performed to compare the metabolites significantly enriched in each season. Red and blue circles represent increase or decrease, respectively, in metabolite abundance during seed germination (a) or dehydration (b), in summer (c) and winter (d), p=0.05. [file 12870_2015_421_MOESM6_ESM.pdf]

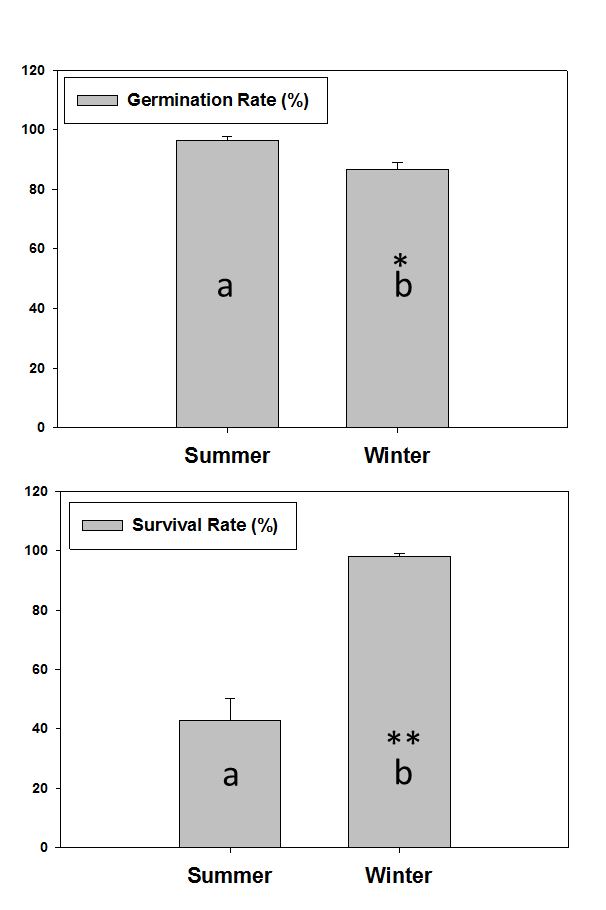

Supplement: Additional file 8: — The average germination and seed survival percentage following dehydration in summer and winter. *p=0.05, **p=0.01. [file 12870_2015_421_MOESM8_ESM.tiff]

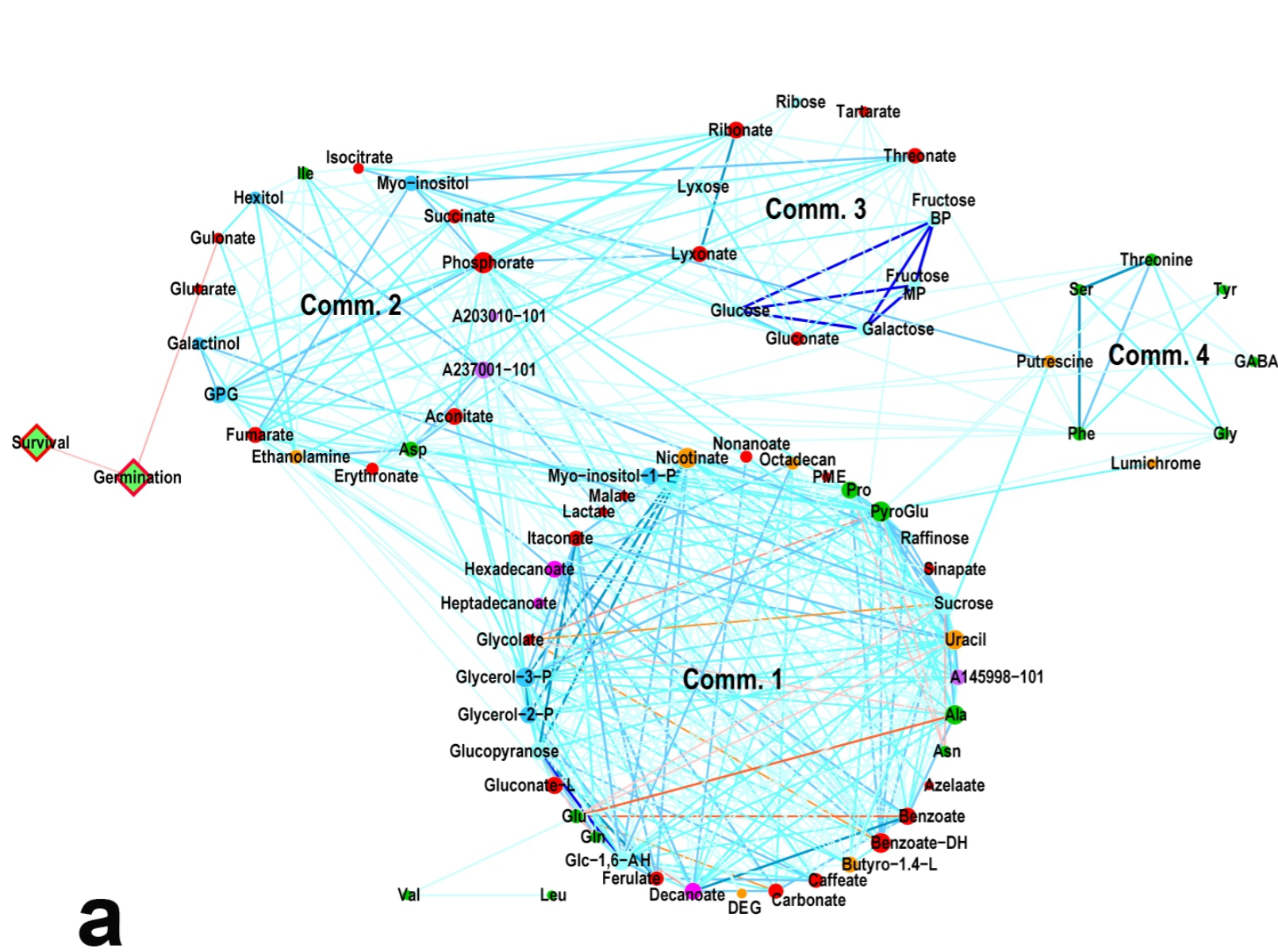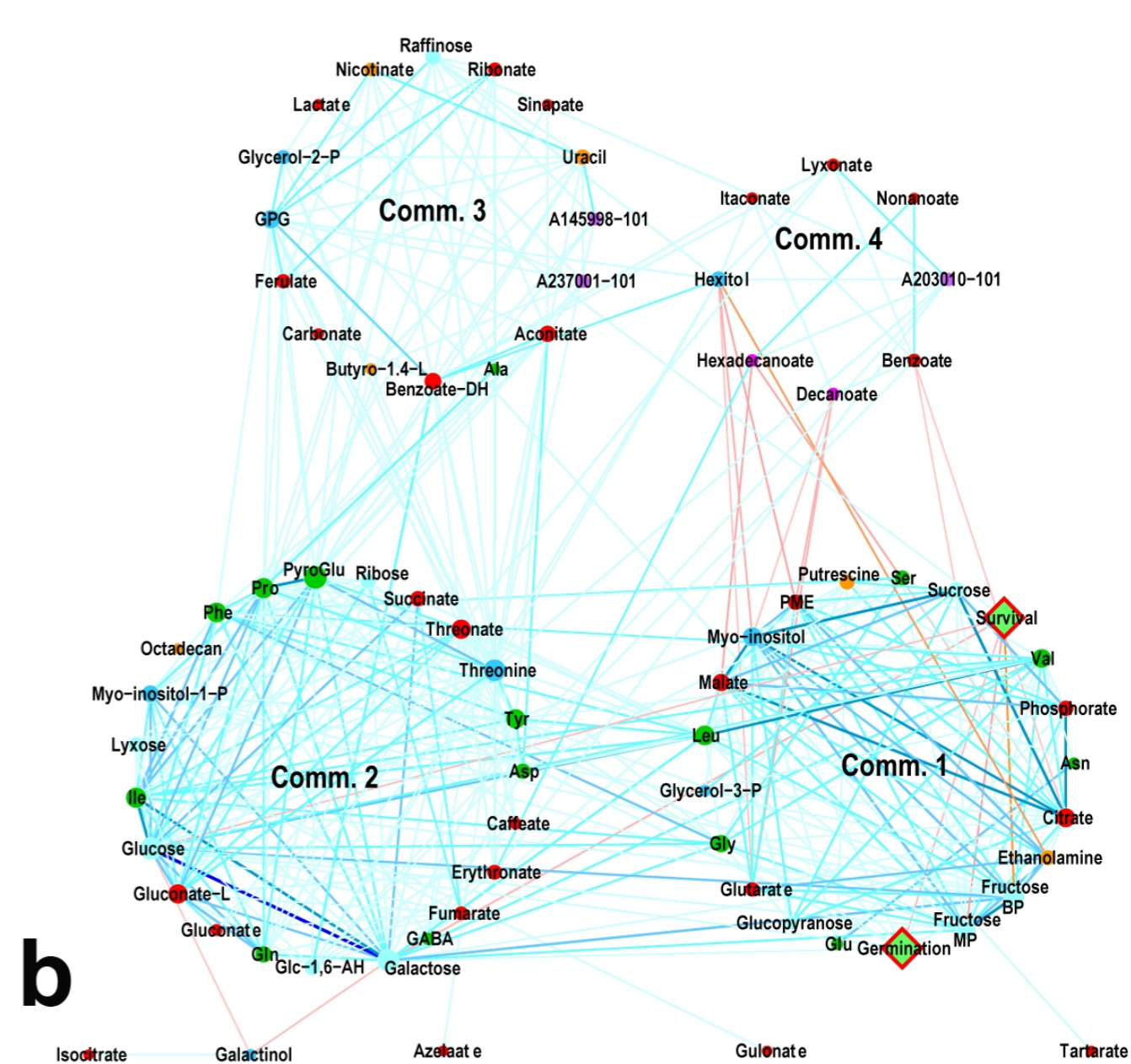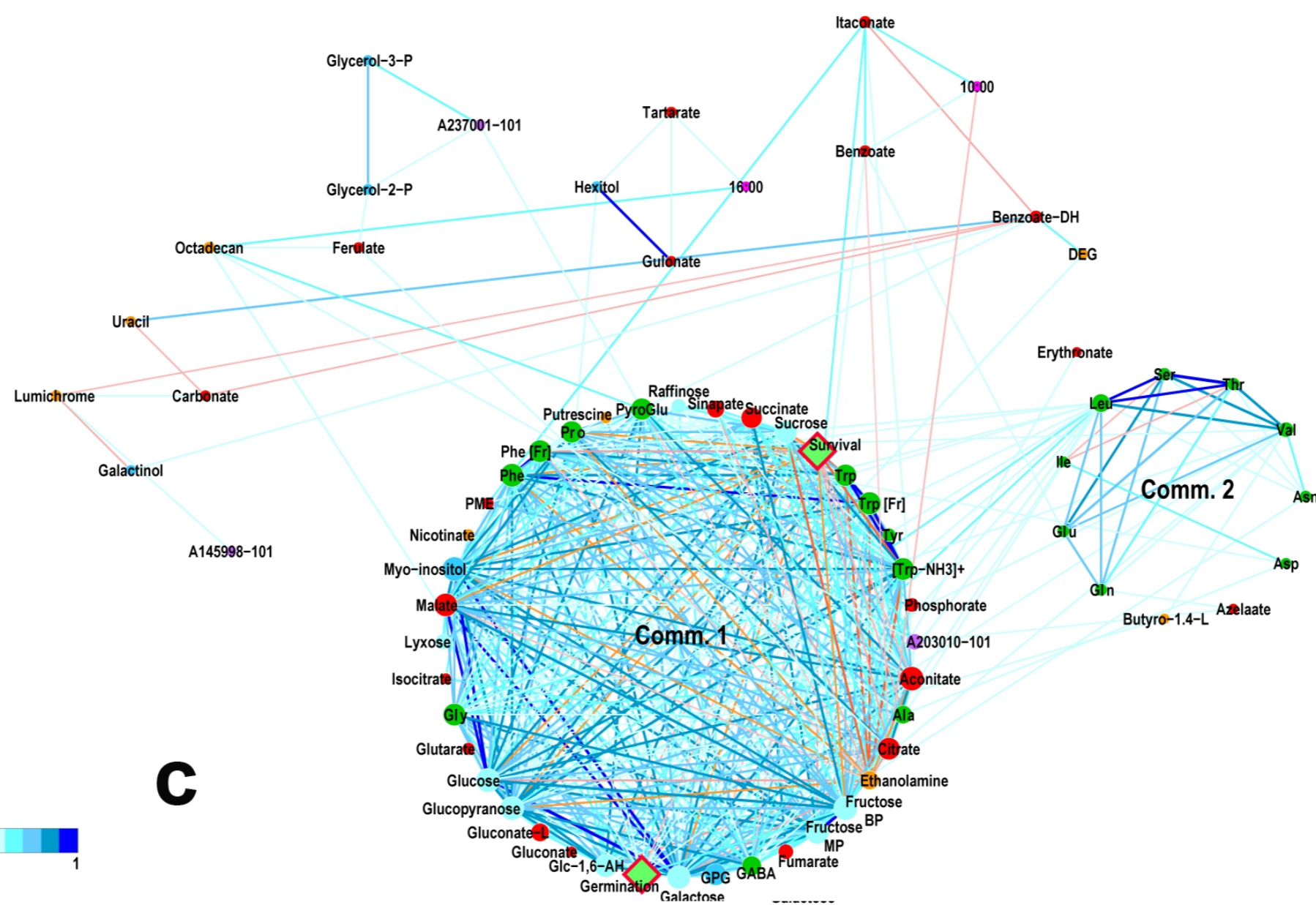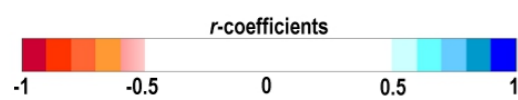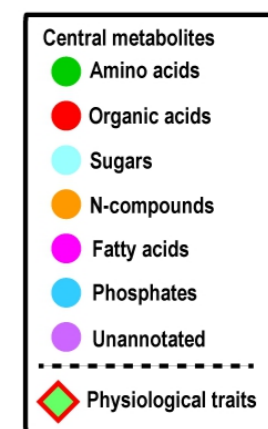

Supplement: Additional file 9: — Network visualization of metabolites as analyzed on dry Shismus arabicus seeds (a), germinated seeds (b), dehydrated seeds (c). Metabolites are clustered according to the walktrap community algorithm. Positive correlations are denoted as blue edges, negative correlations are denoted as red edges. The sizes of the nodes represent the relative degree of connectivity, The widths of edges in the network correspond to the relative magnitude of correlation estimated. [file 12870_2015_421_MOESM9_ESM.pdf]

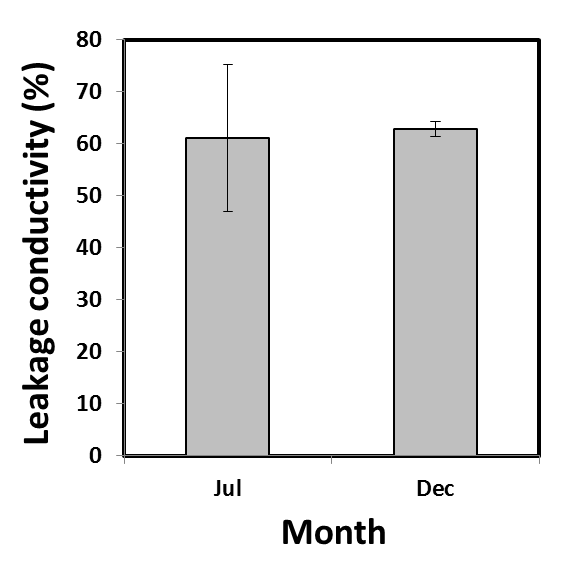

Supplement: Additional file 10: — The dry seed leakage conductivity during the summer and winter months. [file 12870_2015_421_MOESM10_ESM.tiff]
